# Economic Impact of Clinical Pharmacist–Led Medication Review Services in a Psychiatric Hospital for Patients with Somatic Comorbidities: A Cost–Benefit Analysis

**DOI:** 10.1192/j.eurpsy.2026.10417

**Published:** 2026-07-31

**Authors:** M. Stuhec, N. Svetanic, T. Vovk, A. Gorjan Gazdag, Z. Cuk, B. Batinic

**Affiliations:** 1 Department of Pharmacology & Department of Clinical Pharmacy, University of Maribor, Medical Faculty Maribor, Maribor; 2 Department of Clinical Pharmacy, Ormoz’s Psychiatric Hospital, Ormoz; 3 Department of Biopharmaceutics and Pharmacokinetics, University of Ljubljana, Faculty of Pharmacy, Ljubljana, Slovenia; 4 Clinic of Psychiatry, University Clinical Centre of Serbia; 5 Department of Psychology, University of Belgrade, Faculty of Philosophy, Belgrade, Serbia

## Abstract

**Introduction:**

Clinical pharmacists play a critical role in optimising pharmacotherapy through medication reviews in some psychiatric hospitals in patients with mental disorders and somatic comorbidities. Such interventions improve treatment outcomes and enhance patient safety. Although clinical benefits have been proven, there are no economic data on this collaboration in a psychiatric hospital.

**Objectives:**

This study aimed to evaluate the economic impact of clinical pharmacist interventions during medication reviews in a Slovenian psychiatric hospital, including patients with mental disorders and somatic comorbidities, specifically in terms of reducing treatment costs through optimised pharmacotherapy.

**Methods:**

A cost–benefit analysis was conducted to assess the impact of pharmacist interventions in a medication review form during 2013-2018 on treatment cost reduction in the Slovenian psychiatric hospital. Financial values for individual interventions during the medication reviews were obtained from a U.S. study by Lee et al. and adjusted to 2025 values using the CPI Inflation Calculator (Lee et al., Am J Health Syst Pharm 2002; 59 2070–7). The value of other interventions was estimated based on the mean. The cost-benefit ratio between employing a clinical pharmacist and the savings from accepted interventions was calculated. A sensitivity analysis was performed to test robustness with a ±50% variation in estimated savings.

**Results:**

A total of 100 patients were included in the study. Out of 559 recommendations in the medication reviews, 331 were accepted by the psychiatrists, indicating an acceptance rate of 59.2%. The greatest cost savings were achieved through dose adjustments, discontinuation of potentially interacting drugs, and initiation of therapy for untreated conditions. A total of 100 completed medication reviews resulted in a cost reduction of €217,033.6, averaging €2,170.3 per review. The estimated annual cost of employing a clinical pharmacist and delivering 1,200 medication reviews was €160,428.5, while projected annual savings amounted to €2,604,403.6. This corresponds to a return on investment (ROI) ranging from €8.1 to €24.4 per euro invested.

**Conclusions:**

Clinical pharmacist interventions during medication reviews in a psychiatric hospital generate substantial cost savings, with a return of €8.1 to €24.4 for every euro invested, strongly supporting their integration into patient care and positive reimbursement.

**Disclosure of Interest:**

M. Stuhec Grant / Research support from: The author declares that financial support was received for the research and/or publication of this article. The Slovenian Research and Innovation Agency, grant number J3-50127., N. Svetanic: None Declared, T. Vovk: None Declared, A. Gorjan Gazdag: None Declared, Z. Cuk: None Declared, B. Batinic: None Declared

